# Changes in Physical Fitness After 12 Weeks of Structured Concurrent Exercise Training, High Intensity Interval Training, or Whole-Body Electromyostimulation Training in Sedentary Middle-Aged Adults: A Randomized Controlled Trial

**DOI:** 10.3389/fphys.2019.00451

**Published:** 2019-04-24

**Authors:** Francisco J. Amaro-Gahete, Alejandro De-la-O, Lucas Jurado-Fasoli, Manuel Dote-Montero, Ángel Gutiérrez, Jonatan R. Ruiz, Manuel J. Castillo

**Affiliations:** ^1^EFFECTS-262 Research Group, Departament of Medical Physiology, School of Medicine, University of Granada, Granada, Spain; ^2^Promoting Fitness and Health Through Physical Activity Research Group (PROFITH), Department of Physical Education and Sports, Faculty of Sport Sciences, University of Granada, Granada, Spain

**Keywords:** cardiorespiratory fitness, VO_2_max, muscular strength, HIIT, WB-EMS

## Abstract

This study aimed to investigate the influence of different exercise training modalities [(i) a concurrent training based on physical activity recommendation from the World Health Organization group (PAR group), (ii) a high intensity interval training group (HIIT group), and (iii) a high intensity interval training adding whole-body electromyostimulation group (WB-EMS group)] on physical fitness in sedentary middle-aged adults. A total of 89 (52.7% women) middle-aged sedentary adults (53.7 ± 5.1 years old) were enrolled in the FIT-AGING study. Cardiorespiratory fitness was determined by a maximum treadmill test using indirect calorimetry. Lower, upper, and core body muscular strength were assessed by an isokinetic strength test, by the handgrip strength test, and by several core strength endurance tests, respectively. All the exercise types induced similar increases on cardiorespiratory fitness (Δ VO_2_max ≥ 11%, Δ maximal heart rate ≥ 8%, and Δ total test duration ≥ 14%; all P ≤ 0.034), as well as on muscular strength (Δ extension and flexion peak torque ≥ 10%, Δ total hand grip ≥ 3%, Δ core strength endurance tests ≥ 20%; all P ≤ 0.050) compared with a control group. In conclusion, our results suggest that a 12-week structured exercise intervention improves physical fitness regardless of the training program in sedentary middle-aged adults. Despite slightly greater improvements in some physical fitness variables, the changes observed in the WB-EMS group were not superior to the other exercise programs.

## Introduction

Cardiorespiratory fitness (VO_2_max) and muscular strength have been positioned as two independent powerful health markers (Amaro Gahete et al., 2017a). Epidemiological studies have indicated an inverse association of VO_2_max with coronary heart disease, cardiovascular disease events, different types of cancer, and all-cause mortality in both men and women of different ages, which is unaffected by different factors, such as alcohol or tobacco consumption (Kodama et al., 2009; Schmid and Leitzmann, 2015). Furthermore, it has reported that muscular strength is negatively associated with all-cause mortality even after controlling for physical activity levels and VO_2_max (Artero et al., 2012a; Volaklis et al., 2015; Liu et al., 2019).

Several studies have shown that physical exercise is an effective strategy to fight against the high prevalence of chronic diseases (Pedersen and Saltin, 2015), improving physical fitness, and, consequently, increasing quality of life (Fitzgerald et al., 2004; Lee et al., 2010; Volaklis et al., 2015; Harber et al., 2017; Fletcher et al., 2018; García-Hermoso et al., 2018; Ozemek et al., 2018). It is well-known that the application of different training modalities produces important, but not similar health-related physiological adaptations (Huang et al., 2005; Liu and Latham, 2009). The World Health Organization recommended performing concurrent training combining endurance (>150 min/week) and resistance training (>2 sessions/week) (WHO, 2015). Unfortunately, the lack of free time is the principal barrier to do exercise in developed countries (Gómez-López et al., 2010). In this context, alternative and less time-consuming training methodologies that allow us to maximize the potential benefits induced by exercise have recently emerged.

High-intensity interval training has been positioned as an efficient alternative (Gibala et al., 2012) to induce improvements on VO_2_max (Østerås et al., 2005; Osawa et al., 2014; Hwang et al., 2016) and muscular strength (Sculthorpe et al., 2017; Hurst et al., 2018) simultaneously (Hurst et al., 2018), offering potentially better results in older and less fit individuals (Hurst et al., 2018). Although high intensity interval training has been considered the most popular time-efficient exercise methodology, new training tendencies are emerging. Several studies have recently investigated the effects of whole-body electromyostimulation training on health-related parameters (Kemmler et al., 2010, 2014, 2016a,b, 2017, 2018; Von Stengel et al., 2015; Filipovic et al., 2016; Amaro-Gahete et al., 2018a,b). Whole-body electromyostimulation training is a novel training technology that simultaneously innervates up to 12 main muscle groups with a specific electrical intensity. Previous studies have investigated its effects on physical fitness in trained and untrained individuals showing that this training methodology induced a general increase in maximum dynamic and isometric leg-press strength, vertical jump performance, and maximum hand grip strength (Kemmler et al., 2010, 2014, 2016a,b, 2017, 2018; Von Stengel et al., 2015; Filipovic et al., 2016; Amaro-Gahete et al., 2018a,b). Furthermore, an increment in VO_2_max has recently been reported after a 6-week whole-body electromyostimulation training program in recreational runners (Amaro-Gahete et al., 2018a,b).

Little is known about whether different exercise training methodologies could induce different effects on health-related parameters. In this sense, Kemmler et al. (2016a) compared the influence of a high intensity interval training program versus a whole-body electromyostimulation training program. The authors concluded that both training methodologies were equally effective to improve the cardio-metabolic risk profile in sedentary middle-aged men (Kemmler et al., 2016a). However, there are no studies that compare the effects of different exercise training methodologies on physical fitness in sedentary middle-aged adults. Thus, the purpose of this study was to compare the influence of traditional concurrent training vs. high intensity interval training adding or not whole-body electromyostimulation on physical fitness in sedentary middle-aged adults. Our primary hypothesis was that all exercise training programs significantly may improve physical fitness, but that the effects of the high intensity interval training adding whole-body electromyostimulation group (WB-EMS group) could be more significant compared with the traditional concurrent training based on physical activity recommendation from the World Health Organization group (PAR group) and the high intensity interval training group (HIIT group).

## Materials and Methods

### Experimental Approach

A 12-week randomized controlled trial with a parallel group design following the CONSORT (Consolidated Standards of Reporting Trials) guidelines (Schulz et al., 2010) was conducted. For practical and feasibility reasons, the study was conducted in 2 waves with 45 participants maximum. Following the baseline testing (September 2016 and September 2017, respectively), the participants were allocated into four different groups using a computer-generated simple randomization software (Schulz and Grimes, 2002): (i) a concurrent training based on physical activity recommendation from the World Health Organization group (PAR group), (ii) a high intensity interval training group (HIIT group), and (iii) a high intensity interval training adding whole-body electromyostimulation group (WB-EMS group). The randomization process was blinded to the assessment staff. All participants were instructed to maintain their usual physical activity levels and not to engage in other additional structured exercise outside of the intervention program.

### Participants

**FIGURE 1:**
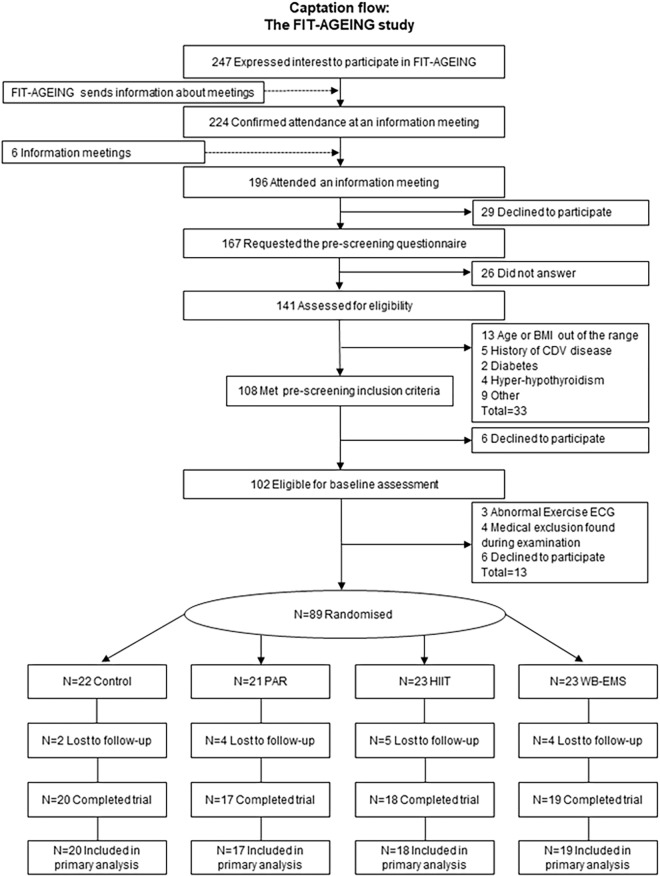
Flow-chart diagram. BMI, body mass index; CDV, cardiovascular; ECG, electrocardiogram; PAR, physical activity recommendations for adults proposed by the World Health Organization group; HIIT, high intensity interval training group; WB-EMS, whole-body electromyostimulation group.

A total of 89 participants (52.7% women) were assessed for eligibility following recruitment via social networks, local media, and posters. Prior to the enrolment, all potential individuals completed a medical examination to identify any pathological condition and current medication that could affect the ability to complete the required exercise training and testing. The inclusion criteria were as follows: (i) adults aged between 40 and 65 years old, (ii) not to be physically active (<20 min of moderate-intensity physical activity on 3 days/week over the previous 3 months), (iii) to have a stable body weight during the previous 6 months (body weight changes <3 kg), and (iv) not to have a history of cardiovascular disease, diabetes mellitus, cancer, and/or major illness (acute or chronic) including any that can limit the ability to complete the necessary exercises. A total of 15 participants dropped out between the randomization and the follow-up due to (i) not having time (*n* = 6), (ii) medical reasons (*n* = 2), (iii) job related relocation (*n* = 3), and (iv) other reasons (*n* = 4). A total of 74 participants were included in the final analysis. All participants provided a written informed consent to participate in the current study^1^ (ID: NCT03334357) (Amaro-Gahete et al., 2018c) which complied with the requirements of the last revised Declaration of Helsinki and was approved by the Human Research Ethics Committee of the “Junta de Andalucía” (0838-N-2017). Figure 1 shows the flow of participants throughout the study.

### Exercise Training Program

A detailed description of each exercise training program can be found elsewhere (Amaro-Gahete et al., 2018c). An attendance of at least 90% of sessions was required to be included in the final analysis. All training sessions were performed in groups of 2–6 participants and a gradual progression was also scheduled in order to ensure a good adherence to each intervention group.

The participants allocated in the PAR group completed 3 concurrent training sessions per week for 12 weeks with at least 48 h of recovery between each session. A total of 150 min/week at 60–65% of the heart rate reserve was established for the endurance training and ∼60 min/week at 40–50% of one-repetition maximum for the resistant training. Different ergometers (i.e., treadmill, cycle-ergometer, and elliptical ergometer) were selected to conduct the endurance training, and weight bearing and guided pneumatic machines were selected to conduct the resistance training (i.e., squat, bench press, dead lift, or lateral pull down).

The participants allocated in the HIIT group completed 2 sessions/week for 12 weeks with at least 72 h of recovery between each session. The participants followed two different and alternative high intensity interval training protocols (Buchheit and Laursen, 2013a,b), which included a high intensity interval training with long intervals protocol (LI) and a high intensity interval training with short intervals protocol (SI). A volume of 40–65 min/week was established at >95% of the maximum oxygen uptake (VO_2_max) in LI, and 6–9 of the ratings of perceived exertion scale (Borg, 1982) in SI. Treadmill with a personalized slope was the exercise modality applied in LI, and 8 weight-bearing exercises (i.e., squat, dead lift, high knees up, high heels up, push up, horizontal row, lateral plank, and frontal plank) in circuit form was the exercise methodology applied in SI.

The participants allocated in the WB-EMS group completed a training program with similar characteristics to those used for the HIIT group adding whole-body electromyostimulation with a wireless device (Wiemspro^®^, Malaga, Spain). The electric pulse was bipolar, symmetrical, and rectangular with a frequency of 15–20 Hz in LI and 35–75 Hz in SI, an intensity of 100 milliamps in SI, and 80 milliamps in SI, an impulse breadth of 200–400 μs in both LI and SI (thighs = 400 μs, glutes = 350 μs, abdominals = 300 μs, low back = 250 μs, mid back = 250 μs, high back = 200 μs, chest = 200 μs, and arms = 200 μs), and a duty cycle (ratio of on-time to the total cycle time: % duty cycle = 100/[total time/on-time]) of 99% in LI and 50–63% in SI, considering previous methodological issues (Amaro Gahete et al., 2017b).

A dynamic standardized warm-up and an active global stretching cooling-down protocol (Amaro-Gahete et al., 2018c) were, respectively, completed at the beginning and at the end of each training session in all intervention groups (Amaro-Gahete et al., 2018c). An extra effort was made to promote maximal attendance. For instance, the sessions were rescheduled when a participant was unable to attend due to work, family, or illness. The participants were constantly motivated throughout each training session and were instructed to reach the specific target intensity. Heart rate was continuously monitored during exercise at 5-s intervals using a pulsometer (Polar RS300, Kempele, Finland).

### Anthropometric and Body Composition Assessment

We measured body weight and height through a pre-validated scale and stadiometer (model 799, Electronic Column Scale, Hamburg, Germany) with light clothes and barefoot. The body mass index was also determined (weight/height^2^).

Body composition was measured using a dual-energy X-ray absorptiometry scanner (Discovery Wi, Hologic, Inc., Bedford, MA, United States). A whole-body scan was used to obtain all parameters. Fat mass index (FMI) and lean mass index (LMI) were calculated as fat mass divided by height^2^ (kg/m^2^) and lean mass divided by height^2^ (kg/m^2^), respectively.

### Dietary Intake Assessment

We performed a total of three 24-h recalls collected on non-consecutive days (one weekend day included) to determine the dietary intake before and after the intervention program (Baugh et al., 2015). Detailed information of the food consumed by the participants was obtained through an interview conducted by qualified nutrition expert. Colored photographs of different food portions sizes were used to help estimate the quantity of food consumed (López and Martín-Lagos, 2010). We used a specific software (EVALFINUT^®^, Ibero-American Foundation of Nutrition, Spain) to calculate energy intake and macronutrient content averaging the three 24-h recalls.

### Sedentary Time and Physical Activity Assessment

Sedentary time and physical activity levels were assessed with a wrist-worn accelerometer (ActiGraph GT3X+, Pensacola, FL, United States) during 7 consecutive days (24 h/day) before and after the intervention (Amaro-Gahete et al., 2018c). The ActiLife v.6.13.3 software (ActiGraph, Pensacola, FL, United States) and the GGIR package (v. 1.5-12^2^) in R (v. 3.1.2^3^) was used to process these files (Hildebrand et al., 2014, 2017). The participants that did not wear the accelerometers for at least 16 h/day during 4 days were discarded.

### Physical Fitness Assessment

A maximum treadmill (H/P/Cosmos Pulsar treadmill, H/P/Cosmos Sport & Medical GMBH, Germany) exercise test following the modified Balke protocol (Balke and Ware, 1959) was used to determine the VO_2_max. We conducted a warm-up (walking at 3.5 km/h for 1 min and at 4 km/h for 2 min) followed by an incremental protocol which started at a speed of 5.3 km/h at 0% grade for 1 min. The grade was then increased 1% every minute until the volitional extenuation of the participants was reached. An indirect calorimeter was used to continuously record the gas exchange (O_2_ consumption and CO_2_ production) using an oronasal mask (model 7400, Hans Rudolph Inc., Kansas City, MO, United States) equipped with a prevent^TM^ metabolic flow sensor (Medgraphics Corp., MN, United States). We performed a flow calibration with a 3-L calibration syringe before the test every day. We calibrated the gas analyzer before each test using two standard gas concentrations. The Breeze Suite software (version 8.1.0.54 SP7, MGC Diagnostic^®^) was used to average O_2_ consumption and CO_2_ production every 5 s. The 6–20 Borg scale (Borg, 1982) was applied to measure the rating of perceived exertion (RPE) at each stage and at exhaustion (during the last 15 s). A familiarization process with the RPE scale was conducted before the exercise test. We continuously recorded heart rate values (Polar RS800, Kempele, Finland) every 5 s. To reach a respiratory exchange ratio ≥ 1.1, a plateau in VO_2_ (change of <100 ml/min in the last 3 consecutive 10-s stages), and a heart rate between 10 beats/min of the age-predicted maximal heart rate (209–0.73 ^∗^ age) (Tanaka et al., 2001) were established as the criteria for achieving VO_2_max. If these criteria were not met, the peak oxygen uptake value during the exercise test was considered (Midgley et al., 2007). The participants were asked to refrain from stimulant substances 24 h before the exercise test, to fast for 3 h, and not to perform any physical activity of moderate (24 h before) and/or vigorous intensity (48 h before).

We used a validated isokinetic strength test (Artero et al., 2012b) on a separate day using a Gymnex Iso-2 dynamometer (EASYTECH s.r.l., Italy) and following the same preconditions established in the maximum treadmill test protocol. We performed a concentric test of both knee flexor and extensor muscles at 60° s^-1^, stabilizing upper members, hips, and shoulders with safety belts. The rotational axis of the dynamometer was aligned with the lateral femoral condyle. We placed the force pad 3–4 cm above the medial malleolus. For safety reasons, we set the knee joint angle between 90 and 170°. We instructed the participants to submaximally flex and extend their knee five times and then to complete three maximal repetitions. A 1-min rest was established between submaximal and maximal trials (Artero et al., 2012b). We determined the flexion and extension peak torque as the single repetition with the highest muscular force output (Nm). We counterbalanced the limb order in the test. The participants were strongly motivated during the test.

A digital hand dynamometer (T.K.K. 5401 Grip-D; Takey, Tokyo, Japan) was used to assess hand grip strength (kg). Two attempts were made for each hand, with a 1-min rest between each trial. We instructed the participants to continuously squeeze for 2–3 s and asked them to exert their maximal force in every attempt. Following previous studies, we fixed the grip spam of the dynamometer at 5.5 cm for men and a validated equation was used for women (Ruiz-Ruiz et al., 2002). We considered total hand grip strength as the sum of best attempt on the left and right hand, respectively.

To assess the core strength performance, we conducted the following four endurance tests: (i) the trunk extensor isometric test, (ii) the trunk flexor isometric test, (iii) the side bridge test (which included both left and right sides), and (iv) the front plank test. The participants were given a minimum of 2 min between efforts to facilitate recovery. In short, the trunk extensor isometric test was modified from the Biering-Sørensen test (Biering-Sørensen, 1984), which has been previously validated as a reliable measure of back extensor performance (McGill et al., 1999). The participants lay prone with the lower body fixed to the test stretcher and keeping their upper bodies on the floor before the exertion. They were instructed to maintain the horizontal position as long as possible, manually recording the endurance time until the upper body came in contact with the floor. The trunk flexor endurance test required the participants to maintain a hip flexion of 60° from the floor, with their knees and hips flexed at 90° (McGill et al., 1999). The test ended when the participants were not able to hold the upper body below the 60° angle. The side bridge test consisted of participants lying on an exercise mat on their sides with their legs extended (McGill et al., 1999). The participants were instructed to lift their hips off the mat and support themselves on one elbow and their feet. The test ended when the hips touched the exercise mat. The front plank test required the participants to assume a prone position with their shoulders and elbows flexed at 90° (McGill et al., 1999). They had to maintain a straight, strong line from head to toes without lowering their hips and keeping their neck in a neutral position with 4 points of support (both forearms and both tiptoes). The test finished when the participants were not able to maintain the correct position.

### Statistical Analyses

Sample size calculations were based on a minimum predicted 15% change in VO_2_max and extension peak torque (with an estimated standard deviation of 15%) between the control group and the exercise groups. Considering the results of a pilot study, 14 individuals per group were necessary to get a statistical power of 85% (type 1 error = 0.05) (Shaffer and Chinchilli, 2007). Nevertheless, a minimum of 20 participants per group were recruited, since a maximum loss of 25% at follow-up was predicted. Data normality was checked using visual check of histograms, Q–Q plots, and the Shapiro–Wilk test.

A repeated-measures analysis of variance was performed to study changes in cardiorespiratory fitness and muscular strength parameters (i.e., VO_2_max in absolute and relative terms, maximal heart rate, total test duration, extension peak torque, flexion peak torque, total hand grip, trunk extensor isometric test, side bridge test, and front plank test) across time, between groups, and the interaction (time^∗^group). Student’s *t*-tests for paired values were applied to determine intragroup differences in cardiorespiratory fitness and muscular strength parameters before and after the intervention study.

We conducted analysis of covariance (ANCOVA) to analyze the effects of the intervention (group entered as fixed factor) on body composition parameters, i.e., post-VO_2_max minus pre-O_2_max (dependent variable), adjusting for the baseline values. The same analyses were conducted for changes in maximal heart rate, total test duration, extension peak torque, flexion peak torque, total hand grip, trunk extensor isometric test, side bridge test, and front plank test. Bonferroni *post hoc* tests with adjustment for multiple comparisons were used to study changes between all exercise groups.

We fixed the level of significance at P < 0.05. The Statistical Package for Social Sciences (SPSS, v. 22.0, IBM SPSS Statistics, IBM Corporation) was used to conduct the statistical analysis and the GraphPad Prism 5 (GraphPad Software, San Diego, CA, United States) to make the graphical plots.

## Results

A total of 74 participants (39 women) were included in the analyses after a loss to follow up of 17% (see Figure 1). We registered an attendance of ∼99, ∼98, and ∼99% of the supervised exercised sessions in the PAR group, the HIIT group, and the WB-EMS group, respectively, from weeks 1 to 12.

The baseline characteristics of all participants and of each separate group are described in Table 1. No differences were observed in the baseline values between groups.

**Table 1:** Descriptive parameters.

	All (*N* = 74)		Control (*N* = 20)		PAR (*N* = 17)		HIIT (*N* = 18)		WB-EMS (*N* = 19)	
	Men (*N* = 35)	Women (*N* = 39)	Men (*N* = 8)	Women (*N* = 12)	Men (*N* = 8)	Women (*N* = 9)	Men (*N* = 9)	Women (*N* = 9)	Men (*N* = 10)	Women (*N* = 9)
Age (years)	54.4 (5.3)	53.0 (5.0)	54.4 (5.3)	53.0 (5.0)	54.5 (5.8)	55.1 (6.1)	52.7 (5.6)	55.8 (4.5)	51.8 (5.4)	51.8 (3.7)
**Body composition**										
Body mass index (kg/m^2^)	28.3 (3.6)	25.3 (3.3)	28.3 (3.6)	25.3 (3.3)	28.9 (1.9)	30.5 (3.8)	25.4 (3.1)	25.1 (2.9)	24.0 (2.1)	26.5 (4.7)
Fat mass (%)	34.7 (8.0)	44.5 (7.4)	34.7 (8.0)	44.5 (7.4)	36.4 (7.5)	38.9 (8.8)	45.3 (8.3)	43.6 (5.7)	45.0 (7.6)	43.8 (8.5)
Fat mass index (kg/m^2^)	10.0 (3.2)	11.4 (2.9)	10.0 (3.2)	11.4 (2.9)	10.6 (2.8)	12.0 (3.7)	11.6 (2.6)	11.0 (2.6)	11.0 (2.7)	11.9 (4.1)
Lean mass (kg)	53.9 (6.5)	34.1 (5.8)	53.9 (6.5)	34.1 (5.8)	56.4 (6.6)	52.8 (7.0)	33.6 (7.4)	35.0 (5.6)	32.5 (4.8)	35.5 (5.1)
Lean mass index (kg/m^2^)	17.5 (2.0)	13.2 (1.8)	17.5 (2.0)	13.2 (1.8)	17.4 (1.6)	17.6 (2.6)	13.1 (2.5)	13.3 (1.4)	12.4 (1.1)	13.8 (1.6)
**Dietary intake**										
Total energy (kcal/d)	2271 (437)	1854 (390)	2408 (356)	1694 (338)	2240 (294)	1853 (371)	2384 (493)	1825 (402)	2107 (497)	2021 (446)
Carbohydrate (g/d)	234.5 (64.6)	195.4 (48.3)	265.2 (94.3)	177.1 (60.3)	229.4 (40.2)	190.4 (33.8)	250.1 (56.0)	199.7 (37.0)	201.8 (56.8)	213.2 (60.5)
Fat (g/d)	96.1 (21.4)	78.8 (22.6)	97.1 (11.8)	77.2 (19.3)	97.3 (28.4)	77.3 (24.3)	98.4 (23.7)	72.8 (26.2)	92.3 (21.8)	87.0 (22.1)
Protein (g/d)	89.2 (22.2)	76.7 (26.5)	82.5 (19.0)	63.4 (11.6)	83.7 (12.0)	85.4 (41.3)	96.5 (24.9)	75.3 (22.1)	90.2 (27.2)	79.9 (15.8)
**Sedentary behavior and PA**										
Valid days (d)	6.7 (0.7)	6.8 (0.9)	6.9 (0.4)	6.5 (1.3)	6.4 (1.1)	6.8 (0.7)	6.9 (0.3)	6.8 (0.7)	6.8 (0.6)	7.1 (0.3)
Wear time (h/d)	24.0 (0.0)	23.9 (0.3)	24.0 (0.0)	23.8 (0.6)	24.0 (0.0)	24.0 (0.0)	24.0 (0.0)	24.0 (0.0)	24.0 (0.0)	23.9 (0.2)
Waking time (h/d)	17.3 (0.7)	16.6 (0.7)	17.2 (0.6)	16.5 (0.9)	17.1 (0.6)	16.6 (0.6)	17.5 (0.7)	16.4 (0.4)	17.2 (0.9)	16.9 (0.6)
Sedentary time (min/day)	770.0 (80.3)	723.7 (82.6)	768.2 (47.0)	733.0 (70.5)	757.2 (111.7)	717.2 (101.2)	797.6 (85.9)	725.7 (62.0)	756.6 (69.3)	716.9 (94.8)
LPA (min/d)	169.6 (49.6)	177.8 (40.9)	165.0 (38.5)	174.3 (39.0)	174.3 (70.3)	179.5 (52.5)	163.1 (52.8)	165.0 (32.4)	175.0 (40.1)	191.7 (39.3)
MPA (min/d)	94.3 (34.9)	94.4 (35.3)	97.7 (19.8)	83.4 (32.3)	90.8 (44.8)	100.0 (46.8)	88.9 (41.6)	90.3 (24.5)	99.7 (32.0)	105.9 (34.8)
VPA (min/d)	2.3 (2.9)	1.1 (1.0)	2.2 (2.4)	0.9 (0.7)	1.4 (1.5)	1.0 (0.7)	3.0 (4.6)	1.4 (1.7)	2.5 (2.5)	1.2 (1.0)
MVPA (min/d)	96.6 (35.5)	95.5 (35.8)	99.9 (18.4)	84.4 (32.8)	92.2 (45.7)	101.0 (47.3)	91.9 (42.1)	91.7 (25.2)	102.2 (33.3)	107.1 (35.4)
Overall PA (ENMO, mG/5 s)	35.8 (8.9)	36.1 (8.8)	36.2 (4.7)	33.8 (8.5)	34.3 (11.8)	37.3 (10.7)	34.9 (10.1)	35.5 (7.9)	37.6 (8.3)	37.9 (8.8)
**Cardiorespiratory fitness**										
VO_2_max (ml/min)	2915 (373)	1809 (332)	2821 (184)	1702 (317)	2795 (494)	1898 (451)	3073 (382)	1850 (283)	2934 (350)	1799 (273)
VO_2_max (ml/kg/min)	33.3 (4.5)	27.9 (5.3)	33.1 (3.3)	26.1 (3.7)	35.0 (6.3)	28.7 (4.4)	33.1 (4.6)	30.1 (7.5)	32.2 (3.6)	27.0 (4.9)
Maximal heart rate (b/min)	162.8 (14.6)	160.5 (13.2)	160.3 (17.8)	155.8 (10.6)	163.9 (11.8)	156.3 (16.0)	159.7 (15.9)	166.6 (11.0)	166.4 (14.3)	163.8 (13.4)
Total test duration (s)	828.2 (182.9)	606.5 (164.1)	845.0 (163.1)	554.0 (169.6)	761.9 (215.4)	552.2 (141.7)	802.8 (152.8)	622.2 (117.6)	892.5 (196.6)	703.3 (193.0)
**Muscular strength**										
Extension peak torque (Nm)	340.2 (67.6)	202.8 (35.4)	314.9 (70.1)	204.3 (39.7)	337.5 (37.7)	212.8 (45.9)	407.7 (52.5)	198.4 (30.3)	297.7 (52.7)	195.3 (24.5)
Flexion peak torque (Nm)	159.1 (43.0)	93.6 (16.6)	146.5 (37.7)	94.7 (17.7)	166.6 (37.8)	95.3 (23.5)	188.1 (49.9)	91.9 (13.6)	134.6 (28.4)	92.2 (11.6)
Total hand grip (kg)	93.1 (12.1)	50.6 (8.2)	91.0 (13.6)	50.8 (8.0)	95.4 (9.8)	51.1 (11.3)	98.2 (11.2)	46.6 (5.0)	88.3 (12.9)	53.9 (6.9)
Trunk extensor isometric test (s)	48.2 (31.2)	52.3 (33.3)	57.4 (45.3)	46.5 (24.5)	43.0 (23.8)	57.2 (44.9)	40.9 (18.4)	51.1 (26.9)	51.7 (34.2)	55.6 (39.6)
Trunk flexor isometric test (s)	157.7 (57.6)	145.2 (59.3)	178.4 (56.9)	155.6 (51.7)	177.1 (44.1)	138.7 (52.8)	133.0 (54.3)	147.5 (57.8)	147.7 (66.5)	136.5 (80.8)
Side bridge test (s)	83.2 (28.3)	61.4 (47.5)	93.0 (15.1)	60.9 (37.7)	88.1 (42.7)	71.4 (81.0)	67.8 (27.0)	41.0 (17.5)	85.6 (19.6)	72.3 (32.9)
Front plank test (s)	56.0 (22.3)	47.0 (26.1)	58.1 (27.1)	49.5 (23.1)	56.4 (25.4)	36.5 (20.8)	54.1 (22.9)	43.5 (22.2)	55.8 (18.4)	57.8 (35.8)


*Data are shown as means ± standard deviation. PAR, physical activity recommendations for adults proposed by the World Health Organization group; HIIT, high intensity interval training group; WB-EMS, whole-body electromyostimulation group; PA, physical activity; LPA, light physical activity; MPA, moderate physical activity; VPA, vigorous physical activity; MVPA, moderate-vigorous physical activity*.

**FIGURE 2:**
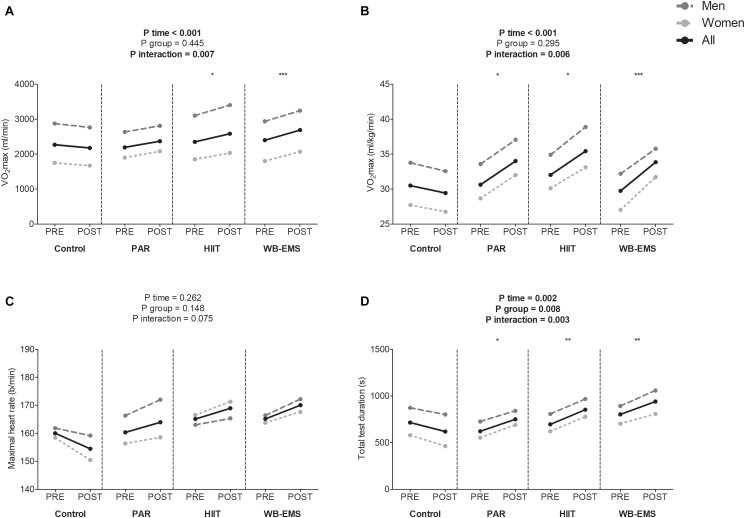
Changes in maximum oxygen uptake (VO_2_max) in absolute **(A)** and relative terms **(B)**, maximal heart rate **(C)**, and total test duration **(D)** values before and after the intervention study. *P*-value [time, group, and interaction (time^∗^group)] of repeated measures analysis of variance. ^∗^*P* < 0.05, ^∗∗^*P* < 0.01, ^∗∗∗^*P* < 0.001 obtained by Student’s paired *t*-test. PAR, physical activity recommendations for adults proposed by the World Health Organization group; HIIT, high intensity interval training group; WB-EMS, whole-body electromyostimulation group.

Figure 2 shows cardiorespiratory fitness-related variables before and after the intervention study. A significant time^∗^group interaction was found in VO_2_max in absolute and relative values, and total test duration (*P* = 0.007, *P* = 0.006, and *P* = 0.003, respectively), whereas a near-significant trend toward significance was observed in the time^∗^group interaction in maximal heart rate (*P* = 0.075). VO_2_max in absolute terms increased in the HIIT group as well as in the WB-EMS group (Δ VO_2_max = 10%; *P* = 0.033, and Δ VO_2_max = 10%; *P* < 0.001, respectively). VO_2_max in relative terms increased in the PAR group as well as in the HIIT group and in the WB-EMS group (Δ VO_2_max = 11%; *P* = 0.026, Δ VO_2_max = 11%; *P* = 0.024, and Δ VO_2_max = 14%; *P* < 0.001, respectively). Total test duration increased in the PAR group as well as in the HIIT group and in the WB-EMS group (Δ Total test duration = 21%; *P* = 0.040, Δ Total test duration = 23%; *P* = 0.003, and Δ Total test duration = 14%; *P* = 0.006). No statistical differences were noted in the control group in any case (all *P* > 0.073).

**FIGURE 3:**
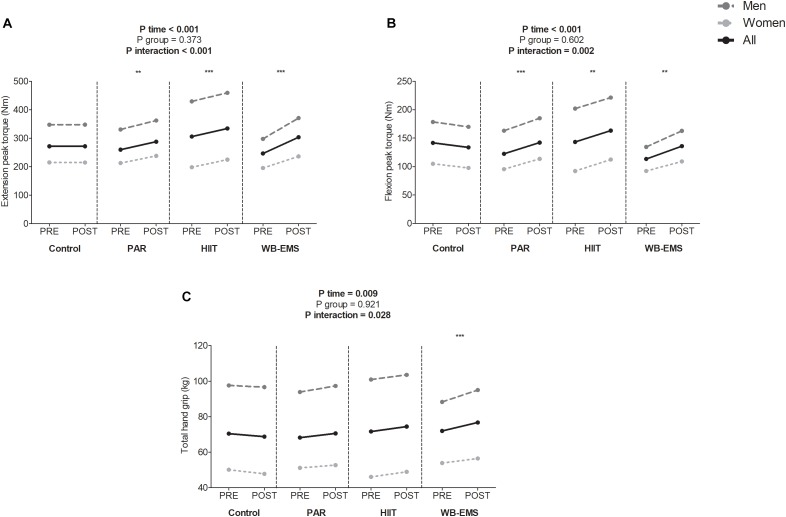
Changes in extension peak torque **(A)**, flexion peak torque **(B)**, and total hand grip **(C)** values before and after the intervention study. *P*-value [time, group, and interaction (time^∗^group)] of repeated measures analysis of variance. ^∗^*P* < 0.05, ^∗∗^*P* < 0.01, ^∗∗∗^*P* < 0.001 obtained by Student’s paired *t*-test. PAR, physical activity recommendations for adults proposed by the World Health Organization group; HIIT, high intensity interval training group; WB-EMS, whole-body electromyostimulation group.

A significant time^∗^group interaction was found in extension peak torque, flexion peak torque, and total hand grip (*P* < 0.001, *P* = 0.002, and *P* = 0.028, respectively; Figure 3). Extension and flexion peak torque increased in the PAR group as well as in the HIIT group and in the WB-EMS group (Δ Extension and flexion peak torque = 11 and 16% for PAR group, Δ Extension and flexion peak torque = 10 and 14% for HIIT group, and Δ Extension and flexion peak torque = 23 and 20% for WB-EMS group, respectively; all *P* ≤ 0.003). Total hand grip increased in the WB-EMS group (Δ Total hand grip = 7%, *P* < 0.001). No statistical differences were noted in the control group in any case (all *P* > 0.270).

**FIGURE 4:**
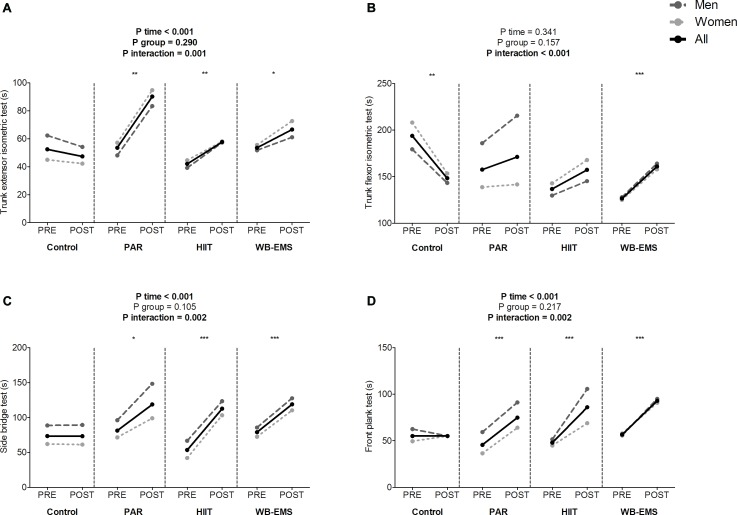
Changes in the trunk extensor isometric test **(A)**, trunk flexor isometric test **(B)**, side bridge test **(C)**, and front plank test **(D)** values before and after the intervention study. *P*-value [time, group, and interaction (time^∗^group)] of repeated measures analysis of variance. ^∗^*P* < 0.05, ^∗∗^*P* < 0.01, ^∗∗∗^*P* < 0.001 obtained by Student’s paired *t*-test. PAR, physical activity recommendations for adults proposed by the World Health Organization group; HIIT, high intensity interval training group; WB-EMS, whole-body electromyostimulation group.

A significant time^∗^group interaction was found in the trunk extensor isometric test, trunk flexor isometric test, side bridge test, and front plank test (*P* = 0.001, *P* < 0.001, *P* = 0.002, and *P* = 0.002, respectively; Figure 4). The trunk extensor isometric test performance increased in the PAR group as well as in the HIIT group and in the WB-EMS group (Δ Trunk extensor isometric test performance = 68%; *P* < 0.001, Δ Trunk extensor isometric test performance = 37%; *P* = 0.003, and Δ Trunk extensor isometric test performance = 24%; *P* = 0.050, respectively). The trunk flexor isometric test performance increased in the WB-EMS group (Δ Trunk flexor isometric test performance = 20%; *P* < 0.001). The side bridge test performance increased in the PAR group as well as in the HIIT group and in the WB-EMS group (Δ Side bridge test performance = 46%; *P* = 0.003, Δ Side bridge test performance = 111%; *P* < 0.001, and Δ Side bridge test performance = 50%; *P* < 0.001, respectively). The front plank test performance increased in the PAR group as well as in the HIIT group and in the WB-EMS group (Δ Front plank test performance = 64% for PAR group, Δ Front plank test performance = 79% for HIIT group, and Δ Front plank test performance = 64% for WB-EMS group; all *P* ≤ 0.001).

**FIGURE 5:**
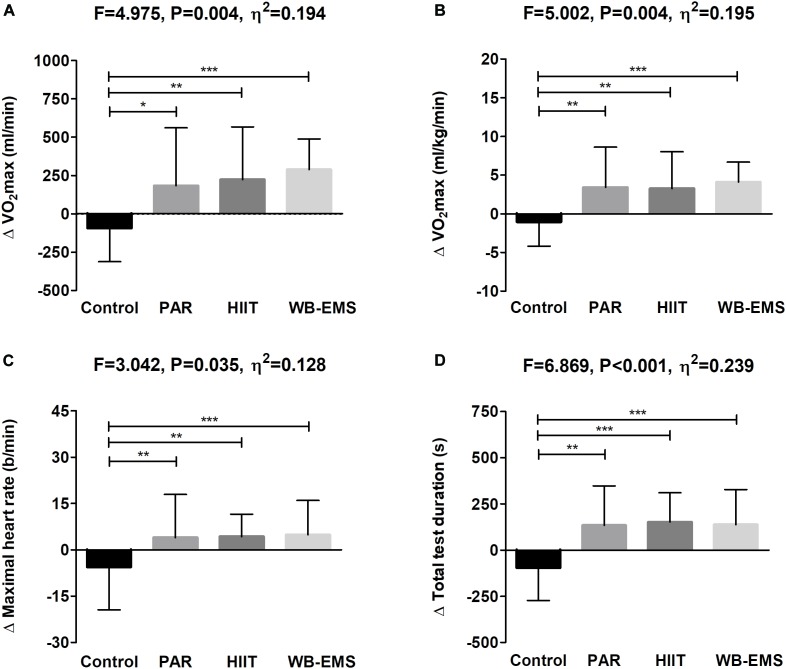
Changes in maximum oxygen uptake (VO_2_max) in absolute **(A)** and relative terms **(B)**, maximal heart rate **(C)**, and total test duration **(D)** after the intervention study among the four groups. Data are shown as means ± standard deviation. Parallel bars indicate significant differences between groups applying an analysis of covariance adjusting by baseline values, with *post hoc* Bonferroni-corrected *t*-test (^∗^*P* < 0.05, ^∗∗^*P* < 0.01, ^∗∗∗^*P* < 0.001). PAR, physical activity recommendations for adults proposed by the World Health Organization group; HIIT, high intensity interval training group; WB-EMS, whole-body electromyostimulation group.

Figure 5 shows changes in the cardiorespiratory fitness-related variables after the intervention study among the 4 groups. The PAR, HIIT, and WB-EMS interventions similarly increased VO_2_max in absolute and relative terms, maximal heart rate, and total test duration compared with the control group (all *P* ≤ 0.034), with no differences between them (all *P* ≥ 0.2). The results persisted in all cases including sex, age, changes in LMI, changes in FMI, changes in energy intake, changes in sedentary time, and changes in overall physical activity levels in the model (see Table 2).

**Table 2:** Changes in physical fitness outcomes adjusted by baseline values (Model 0), by baseline values and sex (Model 1), by baseline values and age (Model 2), by baseline values and changes in lean mass index (Model 3), by baseline values and changes in fat mass index (Model 4), by baseline values and changes in energy intake (Model 5), by baseline values and changes in sedentary time (Model 6), and baseline values and by changes in overall physical activity levels (Model 7).

	Analysis of covariance *P*-value							
	Model 0	Model 1	Model 2	Model 3	Model 4	Model 5	Model 6	Model 7
VO_2_max (ml/min)	**0.006**	**<0.001**	**0.005**	**0.007**	**0.007**	**0.014**	**0.046**	**0.049**
VO_2_max (ml/kg/min)	**0.002**	**0.004**	**0.005**	**0.005**	**0.005**	**0.004**	**0.014**	**0.015**
Maximal heart rate (b/min)	**0.027**	**0.039**	0.053	**0.041**	**0.041**	**0.043**	0.069	0.085
Total test duration (s)	**<0.001**	**<0.001**	**<0.001**	**<0.001**	**<0.001**	**0.001**	**0.001**	**0.002**
Extension peak torque (Nm)	**0.001**	**<0.001**	**0.001**	**0.002**	**0.002**	**0.004**	**0.002**	**0.002**
Flexion peak torque (Nm)	**0.001**	**<0.001**	**0.001**	**0.001**	**0.001**	**0.002**	**0.001**	**0.001**
Total hand grip (kg)	**0.031**	**0.003**	**0.027**	0.127	0.130	0.169	0.098	0.082
Trunk extensor isometric test (s)	**0.004**	**0.010**	**0.010**	**0.016**	**0.018**	**0.022**	**0.009**	**0.007**
Trunk flexor isometric test (s)	**<0.001**	**<0.001**	**<0.001**	**<0.001**	**<0.001**	**<0.001**	**<0.001**	**<0.001**
Side bridge test (s)	**<0.001**	**<0.001**	**<0.001**	**<0.001**	**<0.001**	**<0.001**	**<0.001**	**<0.001**
Front plank test (s)	**0.003**	**0.004**	**0.007**	**0.001**	**0.002**	**0.016**	**0.004**	**0.005**


*Bold type: P < 0.05 (analysis of covariance).*

**FIGURE 6:**
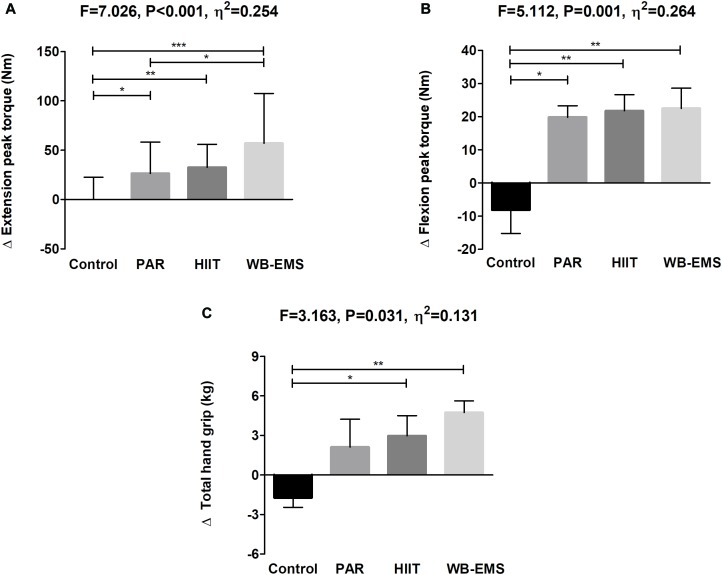
Changes in extension peak torque **(A)**, flexion peak torque **(B)**, and total hand grip **(C)** after the intervention study among the four groups. Data are shown as means ± standard deviation. Parallel bars indicate significant differences between groups applying an analysis of covariance adjusting by baseline values, with *post hoc* Bonferroni-corrected *t*-test (^∗^*P* < 0.05, ^∗∗^*P* < 0.01, ^∗∗∗^*P* < 0.001). PAR, physical activity recommendations for adults proposed by the World Health Organization group; HIIT, high intensity interval training group; WB-EMS, whole-body electromyostimulation group.

Figure 6 shows changes in muscular strength-related variables after the intervention study among the four groups. The PAR, HIIT, and WB-EMS interventions similarly improved extension and flexion peak torque and total hand grip compared with the control group (all *P* ≤ 0.031), with no differences between them (all *P* ≥ 0.1) except when comparing the HIIT vs. the WB-EMS group (*P* = 0.042). The results persisted in all cases when sex, age, changes in LMI, changes in FMI, changes in energy intake, changes in sedentary time, and changes in overall physical activity levels were included as a covariate. This was an exception for total hand grip, in which we observed a partially attenuated effect including changes in LMI, changes in FMI, changes in energy intake, changes in sedentary time, and changes in overall physical activity levels as a covariate (see Table 2).

**FIGURE 7:**
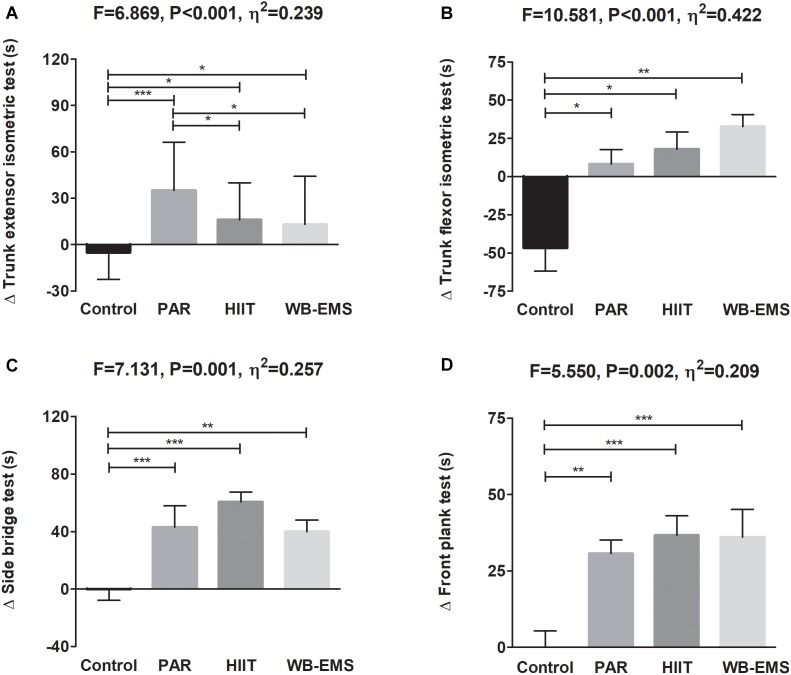
Changes in the trunk extensor isometric test **(A)**, trunk flexor isometric test **(B)**, side bridge test **(C)**, and front plank test **(D)** after the intervention study among the four groups. Data are shown as means ± standard deviation. Parallel bars indicate significant differences between groups applying an analysis of covariance adjusting by baseline values, with *post hoc* Bonferroni-corrected *t*-test (^∗^*P* < 0.05, ^∗∗^*P* < 0.01, ^∗∗∗^*P* < 0.001). PAR, physical activity recommendations for adults proposed by the World Health Organization group; HIIT, high intensity interval training group; WB-EMS, whole-body electromyostimulation group.

Figure 7 shows changes in the core muscular strength-related variables after the intervention study among the four groups. The PAR, HIIT, and WB-EMS interventions similarly increased the trunk extensor and flexor isometric tests, side bridge test, and front plank test performance compared with the control group (all *P* ≤ 0.002). The results persisted when the analyses were additionally adjusted by sex, age, changes in LMI, changes in FMI, changes in energy intake, changes in sedentary time, and changes in overall physical activity (see Table 2).

## Discussion

This study shows that a 12-week structured exercise intervention improves physical fitness regardless of the training program in sedentary middle-aged adults. Despite slightly greater improvements in some fitness variables, the changes observed in the WB-EMS group were not superior to the other exercise programs.

Numerous studies have reported a robust relationship between greater VO_2_max and reduced morbidity and mortality risk, which could indicate that the increment of VO_2_max observed in our study is a significant and clinically relevant finding (Kodama et al., 2009; Schmid and Leitzmann, 2015). Kodama et al. (2009) reported that a 1-unit of metabolic equivalents higher level of cardiorespiratory fitness was associated with a decrement of 13 and 15% in risk of all-cause mortality and cardiovascular disease events, respectively, in healthy men and women (Kodama et al., 2009). In this context, we showed that a 12-week structured exercise intervention increased ∼1 metabolic equivalent irrespective of the training program applied, which is of clinical relevance to quickly and significantly reduce the prevalence of cardiovascular disease events and all-cause mortality.

The absolute increase of VO_2_max in the HIIT group concurred with previous studies (∼8 to 14%) conducted in similar cohorts (Østerås et al., 2005; Osawa et al., 2014; Hwang et al., 2016; Hurst et al., 2018). However, one of these studies compared a 12-week high intensity interval training intervention vs. a 12-week moderate intensity continuous training intervention showing a greater improvement of VO_2_max in response to the first one (Hurst et al., 2018). These results differ from those obtained in our study, since we observed a similar improvement of VO_2_max in both the PAR and HIIT groups. This fact could be explained because we combined endurance with resistance training in the PAR group intervention and a recent metanalysis revealed that a well-designed concurrent training program appears to be beneficial for higher VO_2_max physiological adaptations (Murlasits et al., 2018).

Little is known about the effects of whole-body electromyostimulation on cardiorespiratory fitness. A previous study reported an improvement of maximal aerobic capacity in healthy adults after a 10-week local electromyostimulation training program in quadriceps and hamstring muscles (Nuhr et al., 2003). To the best of our knowledge, there is only two study that investigated the influence of whole-body electromyostimulation on cardiorespiratory fitness suggesting that a 6-week functional and periodized whole-body electromyostimulation training intervention produces an increment of VO_2_max (∼6%) in trained runners despite a considerable reduction of training volume (Amaro-Gahete et al., 2018a,b). These findings concur with those obtained in the current study, but it should be noted that we obtained a larger improvement (∼13%) as a result of having the longest training program duration (6 weeks vs. 12 weeks) and having different training status between these two cohorts (trained runners vs. sedentary middle-aged adults). Although some physiological adaptations that could explain an extra VO_2_max increment after the application of a whole-body electromyostimulation program have been previously described [i.e., (i) a better lower limb coordination and co-activation during exercise, (ii) an increment of the activation capacity of the working muscles during exercise, or (iii) a higher motor unit recruitment and motor unit synchronization, which may induce better mechanical efficiency and motor recruitment actions (Filipovic et al., 2011, 2012)], no significant improvements in the WB-EMS group were noted in our study compared with those obtained in the PAR or the HIIT groups.

It is well-known that muscular strength is negatively and independently associated with all-cause mortality, even controlling by confounder parameters, such as cardiorespiratory fitness, age, or body mass index (Ruiz et al., 2008; García-Hermoso et al., 2018). Therefore, to improve muscular strength during the aging process is of clinical relevance in order to slow down the functional decline and the age-related diseases incidence (Amaro Gahete et al., 2017a). A recent systematic review and metanalysis suggested that concurrent training can impact muscular strength to a greater extent than endurance or resistance training alone (Murlasits et al., 2018). Moreover, Sabag et al. (2018) highlighted that similar increases in muscular strength and hypertrophy were obtained after a concurrent training program compared to a high intensity interval training program including resistance exercise tasks. These findings are consistent with those obtained in our study, since we showed an increase of extension and flexion peak torque and hand grip strength in the PAR group (∼10, ∼15, and 3%, respectively), which concur with the results of previous studies (Takeshima et al., 2004; Eklund et al., 2016; Murlasits et al., 2018). The HIIT group also presented a similar magnitude in our study (∼9, ∼14, and 4%, respectively).

The effects of whole-body electromyostimulation training on muscular strength have been investigated in previous studies (Kemmler et al., 2010, 2014, 2016b, 2017, 2018; Von Stengel et al., 2015; Filipovic et al., 2016; Amaro-Gahete et al., 2018a,b). Their conclusions indicate that this methodology produced significant improvements of: (i) maximum dynamic and isometric leg-press strength in sedentary elderly men (aged >70 years old; ∼9%) (Kemmler et al., 2018), in elite football players (aged ∼25 years old; ∼12%) (Filipovic et al., 2016), in sedentary elderly women (aged >70 years old; ∼10%) (Kemmler et al., 2014), and in postmenopausal sedentary women (aged >70 years old; ∼9%) (Kemmler et al., 2010); (ii) vertical jump performance in recreational runners (aged ∼27 years old; ∼8%) (Amaro-Gahete et al., 2018a), and in elite football players (aged ∼25 years old; ∼10%) (Filipovic et al., 2016); (iii) maximum hand grip strength in sedentary elderly men (aged >70 years old; ∼6%) (Kemmler et al., 2017) and in sedentary elderly women (aged >70 years old; ∼8%) (Von Stengel et al., 2015; Kemmler et al., 2016b). Our results concur with previous long-term studies, since we showed a significant increase of extension and flexion peak torque and hand grip strength in the WB-EMS group (∼23, ∼19, and 6%, respectively). This might be explained because (i) we conducted a functional and periodized high intensity interval training program adding whole-body electromyostimulation following the recommendations provided by Filipovic et al. (2011) in terms of electrical parameters (impulse frequency, impulse intensity, impulse width, and duty cycle) to effectively improve muscular strength. Most previous studies, however, used a pre-determined training methodology based on isometric weight-bearing exercises (1–2 sets of 8 repetitions) and applied an impulse frequency of 85 Hz, an impulse width of 350 μs, and a duty cycle of 50% (Kemmler et al., 2010, 2014, 2016a,b, 2017, 2018; Von Stengel et al., 2015). (ii) The participant’s characteristics of our study were different than in other studies (i.e., sex, age, training status, etc.).

Moreover, although a previous study compared the influence of a high intensity interval training program vs. a whole-body electromyostimulation training program on cardio-metabolic risk factor in sedentary men (Kemmler et al., 2016a), there are no studies that compare the effects of these training methodologies on muscular strength in sedentary middle-aged adults applying the same exercises and training loads approach. Our results revealed that, although no significant differences were obtained in muscular strength-related parameters, clinically relevant improvements were noted in the WB-EMS compared to the HIIT group in extension and flexion peak torque and hand grip strength (∼23% vs. ∼9%; ∼19% vs. ∼14%; and 6% vs. 4%, respectively). Therefore, our findings suggest that a whole-body electromyostimulation training, as a novel stimulus, could complement the traditional high intensity interval training structure enhancing muscular strength in sedentary middle-aged adults.

## Limitations

Our study had a number of limitations. Firstly, the sample size was relatively small to study the influence of these different exercise training interventions on physical fitness considering both sexes separately, although no interaction effects were observed. Considering that we compared a total of three different exercise training programs, our study could be underpowered to note statistical differences in specific physical fitness-related parameters between them. Moreover, although the results remained after adjusting the analysis for some confounder variables, further trials involving a greater number of participants are needed to accurately determine training induced changes when comparing these three exercise methodologies. Finally, the results of the present study are representative of a sedentary healthy adult population aged between 40 and 65 years old, and therefore might not be extrapolated to active, younger, or older adults, including those with acute or chronic diseases.

## Conclusion

Our findings suggest that a 12-week structured high intensity interval training program adding whole-body electromyostimulation did not significantly improve overall physical fitness compared with both a high intensity interval training program without whole-body electromyostimulation and a traditional concurrent training program in sedentary middle-aged adults. However, clinically relevant improvements were observed in the WB-EMS group in some physical fitness variables. Therefore, further studies with greater sample size and longer duration are needed to elucidate whether the combination of voluntary exercise and whole-body electromyostimulation is effective to increase both cardiorespiratory fitness and muscular strength.

## Ethics Statement

All participants provided a written informed consent to participate in the current study (http://www.clinicaltrials.gov, ID:NCT03334357) (Amaro-Gahete et al., 2018c) which complied with the requirements of the last revised Declaration of Helsinki and was approved by the Human Research Ethics Committee of the “Junta de Andalucía” (0838-N-2017). Figure 1 shows the flow of participants throughout the study.

## Author Contributions

FA-G, AD-l-O, ÁG, JR, and MC conceived and designed the study. FA-G, AD-l-O, LJ-F, and MD-M performed the tests and the intervention training. FA-G and JR performed the statistical analysis. FA-G drafted the manuscript. ÁG, JR, and MC revised the manuscript. All authors read and approved the final manuscript.

## Conflict of Interest Statement

The authors declare that the research was conducted in the absence of any commercial or financial relationships that could be construed as a potential conflict of interest.

## Footnotes

1 http://www.clinicaltrials.gov

2 https://cran.r-project.org/web/packages/GGIR/

3 https://www.cran.r-project.org/
